# Elabela Protects Spontaneously Hypertensive Rats From Hypertension and Cardiorenal Dysfunctions Exacerbated by Dietary High-Salt Intake

**DOI:** 10.3389/fphar.2021.709467

**Published:** 2021-07-27

**Authors:** Xavier Sainsily, David Coquerel, Hugo Giguère, Lauralyne Dumont, Kien Tran, Christophe Noll, Andrei L. Ionescu, Jérôme Côté, Jean-Michel Longpré, André Carpentier, Éric Marsault, Olivier Lesur, Philippe Sarret, Mannix Auger-Messier

**Affiliations:** ^1^ Département de Médecine, Centre de Recherche du CHUS, Faculté de Médecine et des Sciences de la Santé, Université de Sherbrooke, Sherbrooke, QC, Canada; ^2^ Département de Pharmacologie et Physiologie, Centre de Recherche du CHUS, Faculté de Médecine et des Sciences de la Santé, Université de Sherbrooke, Sherbrooke, QC, Canada; ^3^ Institut de Pharmacologie de Sherbrooke, Université de Sherbrooke, Sherbrooke, QC, Canada

**Keywords:** Elabela/Toddler, Apelin, APJ receptor, ACE2, neprilysin, hypertension, renin–angiotensin–aldosterone system, sodium diet

## Abstract

**Objectives:** Arterial hypertension, when exacerbated by excessive dietary salt intake, worsens the morbidity and mortality rates associated with cardiovascular and renal diseases. Stimulation of the apelinergic system appears to protect against several circulatory system diseases, but it remains unknown if such beneficial effects are conserved in severe hypertension. Therefore, we aimed at determining whether continuous infusion of apelinergic ligands (i.e., Apelin-13 and Elabela) exerted cardiorenal protective effects in spontaneously hypertensive (SHR) rats receiving high-salt diet.

**Methods:** A combination of echocardiography, binding assay, histology, and biochemical approaches were used to investigate the cardiovascular and renal effects of Apelin-13 or Elabela infusion over 6 weeks in SHR fed with normal-salt or high-salt chow.

**Results:** High-salt intake upregulated the cardiac and renal expression of APJ receptor in SHR. Importantly, Elabela was more effective than Apelin-13 in reducing high blood pressure, cardiovascular and renal dysfunctions, fibrosis and hypertrophy in high-salt fed SHR. Unlike Apelin-13, the beneficial effects of Elabela were associated with a counter-regulatory role of the ACE/ACE2/neprilysin axis of the renin-angiotensin-aldosterone system (RAAS) in heart and kidneys of salt-loaded SHR. Interestingly, Elabela also displayed higher affinity for APJ in the presence of high salt concentration and better resistance to RAAS enzymes known to cleave Apelin-13.

**Conclusion:** These findings highlight the protective action of the apelinergic system against salt-induced severe hypertension and cardiorenal failure. As compared with Apelin-13, Elabela displays superior pharmacodynamic and pharmacokinetic properties that warrant further investigation of its therapeutic use in cardiovascular and kidney diseases.

## Introduction

The apelinergic system is involved in numerous physiological functions, such as fluid homeostasis, energy metabolism as well as regulation of cardiovascular development and function (Pitkin et al., 2010; Kidoya et al., 2015; Hwangbo et al., 2017; Kuba et al., 2019). It is composed of the endogenous ligands Apelin (which include several active fragments Apelin-13, Apelin-17, Apelin-36) and Elabela as well as of the APJ receptor, a class AG protein-coupled receptor first discovered in 1993 based on its homology with the angiotensin II type 1 receptor (AT1R) (O’Dowd et al., 1993; Ma et al., 2017). Over the last decade, the APJ receptor has received ongoing interest as an attractive therapeutic target for the treatment of heart failure and cardiovascular diseases, notably due to its inodilator properties following activation, causing marked arterial vasodilation and positive inotropic effect (Tatemoto et al., 1998; Szokodi et al., 2002; Coquerel et al., 2018). Importantly, administration of [Pyr^1^]-Apelin-13 (Ape13), the predominant Apelin fragment detected in human plasma and heart (Maguire et al., 2009; Zhen et al., 2013), was found to exert beneficial effects in heart failure patients (Japp et al., 2010). Discovered more recently, Elabela-32 has been shown to mediate self-renewal of human embryonic stem cells (Ho et al., 2015), to regulate endoderm differentiation, and to contribute to cardiovascular development during zebrafish and mouse embryogenesis (Chng et al., 2013; Ho et al., 2017). Moreover, Elabela-32 increases the contractility of *ex-vivo* isolated rat heart at adulthood (Perjes et al., 2016; Coquerel et al., 2017). Since then, sustained activation of the Ape13-Elabela-APJ receptor axis has demonstrated significant cardio-protective effects in various rodent models of cardiac dysfunctions (Chagnon et al., 2017; Coquerel et al., 2017; Ho et al., 2017; Sato et al., 2017; Yang et al., 2017; Coquerel et al., 2018).

Hypertension is a multifactorial disease resulting from genetic and environmental factors that affect the cardiovascular and renal functions. It is responsible for a striking 40% of the morbidity and mortality associated with cardiovascular diseases (Basting and Lazartigues, 2017). In human, hypertension is manifested by a systemic elevated arterial blood pressure leading to heart failure, cerebrovascular diseases and kidney damage (Sekerci et al., 2018). Indeed, several clinical studies indicate a correlation between the progression of renal failure with the increased of blood pressure (Wright et al., 2002; Chobanian et al., 2003). Moreover, excessive dietary salt intake on a chronic basis may result in an increase in proteinuria and elevation of pressure overload in salt-sensitive individuals, leading to cardiac dysfunction and progressive renal injury (Du Cailar et al., 2004; Williams et al., 2006). The spontaneously hypertensive rat (SHR) model closely mimics human hypertension which progresses toward heart failure with pathophysiological changes associated with cardiac and renal dysfunction (Ferrone et al., 1979; Doggrell and Brown, 1998). As observed in humans, cardiac and renal damage is aggravated in these animals when a high-salt intake regimen is administered (Doggrell and Brown, 1998). It has been postulated that the pathophysiological, structural, and functional changes induced by chronic salt loading could be mediated through the action of the renin-angiotensin-aldosterone system (RAAS) in the vasculature, heart, and kidneys (Frohlich, 2008). In this study, we thus investigated whether the apelinergic system could provide beneficial effects to SHR rats fed with normal-salt or high-salt diet. We demonstrated here that Elabela-32 exerts potent and greater cardiorenal protective effects than Ape13 in rodents with severe salt-induced hypertension.

## Materials and Methods

### Animals and Experimental Protocols

Male spontaneously hypertensive rats (SHR) and normotensive Wistar-Kyoto (WKY) rats were purchased from Charles River Laboratories (Kingston, ON, Canada) and maintained in a temperature-controlled room with a 12 h light/dark cycle at the animal facility of the Université de Sherbrooke. All animals were randomized with unique identification number into the different groups tested to maintain experimenter blinding. The study included six distinct SHR experimental groups (Table 1). Three groups received a standard rat chow diet containing 0.3% of NaCl (Charles River, ON, Canada) while the other three groups were fed a rat diet containing 8% of NaCl for 6 weeks (Research Diets, NJ, United States). Once a week, urine output and water intake were measured over 24 h using metabolic cages following 3 days of acclimatization. After 6 weeks of different salt intake regimens, the rats were anesthetized with 2% isoflurane for echocardiographic and hemodynamic measurements. Subsequently, blood samples were collected into tubes containing K2-EDTA and centrifuged at 2000 g for 10 min at 4°C before storing the plasma at −80°C. Plasma and urinary concentrations of sodium were determined by a vitros 750 XRC analyser (Johnson-Johnson Clinical Diagnostics; Rochester, NY). OCT-embedded sections of heart (7 µm) were generated and collagen fibers visualized by Masson’s trichrome staining. Immunohistochemistry stained slides were captured by an automated Hamamatsu Nanozoomer 2.0RS slide scanner (Hamamatsu Photonics). Heart and kidneys were also harvested, snap frozen in liquid nitrogen and stored at −80°C for mRNA expression analysis. All animal procedures were approved by our institutional ethical committee (protocol #2017–2115), in compliance with the policies and directives of the *Canadian Council on Animal Care* and with the ARRIVE guidelines.

**TABLE 1:** Effect of apelinergic ligands on heart function and hemodynamics in SHR rats. Mean, systolic and diastolic arterial blood pressure (AP) were measured with a pressure catheter. Echocardiography measurement of heart rate (HR), aortic pulse propagation velocity (APPV), fractional shortening (FS%), left ventricular end systolic (LVESD) and diastolic (LVEDD) diameters, anterior (AWTS) and posterior (PWTS) wall thickness during systole were determined after 6 weeks of diet with normal-salt or high-salt chow, and treated with either saline, Apelin-13 (Ape13) or Elabela (ELA). All values are means ± SEM, the data were analyzed with one-way ANOVA and Bonferroni’s post hoc test. *p < 0.05, **p < 0.01 vs SHR fed with normal-salt chow.

	SHR rats					
	Normal-salt diet			High-salt diet		
	Saline	Ape13	ELA	Saline	Ape13	ELA
n	5	5	5	6	6	6
Weight (g)	326 ± 7	321 ± 5	333 ± 7	332 ± 7	339 ± 10	320 ± 5
Mean AP (mmHg)	147 ± 8	143 ± 14	149 ± 12	185 ± 17	152 ± 19	112 ± 15*
Systolic AP (mmHg)	183 ± 12	179 ± 19	186 ± 16	234 ± 19	194 ± 23	141 ± 16**
Diastolic AP (mmHg)	129 ± 6	125 ± 12	131 ± 11	163 ± 13	131 ± 17	97 ± 14**
*Echocardiography*						
HR (bpm)	352 ± 15	372 ± 4	332 ± 5	370 ± 9	348 ± 12	333 ± 13
APPV (m/s)	4.7 ± 0.2	4.5 ± 0.4	3.5 ± 0.3	4.6 ± 0.2	4.6 ± 0,3	3.2 ± 0.2**
FS (%)	38.8 ± 3.3	39.8 ± 1.1	39.5 ± 0.7	32.4 ± 3.3	37.6 ± 3.3	45.8 ± 2.1*
LVESD (mm)	4.4 ± 0.4	3.8 ± 0.3	4.1 ± 0.1	4.7 ± 0.4	4.5 ± 0.3	3.4 ± 0.2*
LVEDD (mm)	6.7 ± 0.3	6.3 ± 0.4	6.8 ± 0.1	7.0 ± 0.3	7.2 ± 0.1	6.8 ± 0.2
AWTS (mm)	2.4 ± 0.1	2.3 ± 0.1	2.1 ± 0.1	2.6 ± 0.1	2.4 ± 0.1	2.0 ± 0.1**
PWTS (mm)	2.6 ± 0.2	2.3 ± 0.1	2.2 ± 0.1	2.8 ± 0.1	2.6 ± 0.1	2.1 ± 0.1**

### Peptide Synthesis and Administration

[Pyr^1^]-Apelin-13 (hereafter referred as Ape13; molecular weight: 1,533.82 g/mol) is synthesized at 0.1 mmol scale using solid phase peptide synthesis, as previously described (Murza et al., 2015). Briefly, 2-chlorotrityl chloride resin (2-CTC, 120 mg) was loaded with Fmoc-L-Phe-OH (0.85 mmol/g). Fmoc protecting group was removed by treating the resin with a 20% piperidine solution. The coupling steps were carried out using [hexafluorophosphate of O-(7-azabenzotriazol-1-yl)-1,1,3,3-tetramethyluronium] (HATU, 5 equiv), amino acid (5 equiv) and DIPEA (5 equiv). The final peptide was cleaved from resin using a cocktail of trifluoroacetic acid (TFA)/triisopropylsilane (TIPS)/ethanedithiol (EDT)/water (92.5/2.5/2.5/2.5) and purified by preparative HPLC (ACE5 C18 column 250 × 21.2 mm, 5 μm spherical particle size). The linear precursor peptide of human Elabela-32 (hereafter referred as Elabela; molecular weight: 3,950.79 g/mol) was synthesized with the same protocol mentioned above (2-CTC, 500 mg, loading 0.2 mmol/g). After peptide cleavage, the disulfide bridge was made using a solution of 10% Iodine/MeOH (dropwise until persistent yellow color observed). Finally, peptide was purified by preparative HPLC. Purity (>99%) and authenticity of both peptides were confirmed by UPLC-MS and HRMS. Previously, we have shown from *in vitro* competitive binding assays that Ape13 and Elabela displaced the radioligand [Glp65, Nle75, Tyr77][125I]-Apelin-13 with comparable affinity (Ki = 0.37 ± 0.04 and 0.19±0.02 nM, respectively) for the APJ receptor (Murza et al., 2016). This led us to test an equimolar dose (10 nmol/kg/hr) of Ape13 and Elabela (i.e., 15 μg/kg/hr and 39 μg/kg/hr, respectively) in a cecal-ligation puncture model of *in vivo* experimental sepsis induced in adult male Sprague-Dawley rats (Coquerel et al., 2017). In this previous study, we showed that Ape13 improved the survival of these rats to the same extent than Elabela. Therefore, the rationale for this study was to administer by subcutaneous infusion, using osmotic pumps (Alzet model 2006, Alza Corp., CA, United States), the same equimolar dose (10 nmol/kg/hr) of Ape13 and Elabela in male 10-week-old SHR rats fed for 6  weeks with normal-or high-salt diets, as these doses were shown to be effective in experimental *in vivo* sepsis models.

### Echocardiography and Invasive Hemodynamics

Transthoracic echocardiography was performed under isoflurane anesthesia (2%; 1.5 ml/min; Baxter) with the Vevo 3,100 ultrasound apparatus using a MX250 transducer (FUJIFILM VisualSonics, ON, Canada). A short axis view of the left ventricle (LV) was obtained at the level of the papillary muscle and the M-mode tracing was recorded. LV End-Diastolic and End-Systolic Diameters (LVEDD; LVESD) were measured in order to calculate the fractional shortening (FS). Stroke volume was next calculated in order to assess Cardiac Output (CO) and Cardiac Index (CI). Pulse propagation velocity (PPV) was determined by ultrasound measurements of the abdominal aorta using EKV-image acquisition and analyzed with Vevo-Vasc software (FUJIFILM VisualSonics, ON, Canada). For invasive hemodynamic measurements, rats were intubated under isoflurane anesthesia (2%; 1.5 ml/min; Baxter, IL, United States) and ventilated (SAR-1000, CWE). The right carotid artery was then cannulated with a pressure catheter (1.9F, Transonic, NY, United States) and advanced into the LV to record end-systolic and end-diastolic pressures.

### Quantitative Reverse Transcription PCR

Total RNA was extracted from heart and kidneys using RNeasy Mini kits (Qiagen) according to the manufacturer’s protocol. iScript Reverse Transcription Supermix (Bio-Rad) was used to prepare cDNA from 1 µg of total RNA in 20 µL. Real-time PCR was performed on technical duplicates with cDNA diluted 30x in nuclease-free water and using SsoAdvanced™ Universal SYBR® Green Supermix (Bio-Rad) in a Mastercycler® ep RealPlex (Eppendorf). The Ribosomal protein L30 (Rpl30) of heart or kidney was used as a reporter gene and fold changes were obtained using the 2(-ΔΔCt) method (Livak and Schmittgen, 2001). Data were then normalized to SHR rats receiving a standard chow diet containing 0.3% of NaCl. Primer sequences are given in Table 2.

**TABLE 2:** Sequences of the primers (forward: Fwd; reverse: Rev) used to determine by quantitative RT-PCR the expression level of target genes (Gene ID) identified by their corresponding mRNA reference sequence accession number (mRNA RefSeq).

Gene ID (mRNA RefSeq)	Gene description	Sequences (5'->3′)
*Rpl30* (NM_022699.4)	Ribosomal protein L30	Fwd: TCT​TGG​CGT​CTG​ATC​TTG​GT
		Rev: AAG​TTG​GAG​CCG​AGA​GTT​GA
*Apln* (NM_031612.3)	Apelin	Fwd: CAT​GGC​CTC​TTC​CTT​AGC​TC
		Rev: CAG​CGG​CAA​TAG​AAC​AGA​GA
*Ela* (XM_039095067.1)	Elabela	Fwd: ACT​TCA​TTC​TCG​AGT​GCC​CTT​C
		Rev: TGG​ATC​CGA​AAA​GCC​ATC​CAA
*Aplnr* (NM_031349.2)	Apelin receptor	Fwd: CTT​CTA​GGC​ACC​ACA​GGC​AA
		Rev: GAG​CGT​CTC​TTT​TCT​CGG​CT
*Tgfb2* (NM_031131.2)	Transforming growth factor beta 2	Fwd: GAC​TTA​CTG​CAG​GAG​AAG​GCA​A
		Rev: CAC​TGA​GCC​AGA​GGA​TGT​TGT​A
*Col1a1* (NM_053304.1)	Collagen type I alpha 1 chain	Fwd: GTA​CAT​CAG​CCC​AAA​CCC​CA
		Rev: CAG​GAT​CGG​AAC​CTT​CGC​TT
*Acta1* (NM_019212.3)	Actin, alpha 1, skeletal muscle	Fwd: AGG​ACC​TGT​ACG​CCA​ACA​AC
		Rev: GGG​TGC​GCC​TAG​AAG​CAT​T
*Ace* (NM_012544.1)	Angiotensin I converting enzyme	Fwd: TTT​GCT​ACA​CAA​ATG​GCA​CTT​GT
		Rev: CGG​GAC​GTG​GCC​ATT​ATA​TT
*Ace2* (NM_001012006.2)	Angiotensin I converting enzyme 2	Fwd: CGC​TGT​CAC​CAG​ACA​AGA​A
		Rev: CGT​CCA​ATC​CTG​GTT​CAA​G
*Mme* (NM_012608.2)	Neprilysin	Fwd: CAA​ACA​CAA​ACT​CTG​GGG​TGA​G
		Rev: AGG​ACG​CAT​GGA​GTA​ACA​CA

### Radioligand Binding

HEK293 cells stably expressing the YFP epitope-tagged human APJ were washed once with PBS and subjected to one freeze-thaw cycle. Broken cells were gently scraped in resuspension buffer (10 mM Tris-HCl, pH 7.5, 1 mM EDTA), centrifuged at 3,500 g for 15 min at 4°C and resuspended in binding buffer (50 mM Tris-HCl, pH 7.5, 0.2% bovine serum albumin, containing 140 mM or 330 mM NaCl). For total heart binding experiments, tissues were minced in extraction buffer (250 mM sucrose, 1 mM EDTA, 2 mM EGTA, 1x cocktail of protease and phosphatase inhibitors from ThermoFisher), centrifuged at 500 g for 5 min at 4°C and the supernatant was centrifuged at 10,000 g for 30 min before resuspension in binding buffer (50 mM Tris-HCl, pH 7.5, containing 0.2% bovine serum albumin). Competitive radioligand binding experiments were performed by incubating cell membranes (15 µg) or total heart extraction (500 µg) with 0.2 nM [Glp^65^, Nle^75^, Tyr^77^][^125^I]-Apelin-13 (820 Ci/mmol) and increasing concentrations of Ape13 or Elabela (10^−11^ to 10^−5^ M) for 1 h at room temperature in a final volume of 200 µL. Bound radioactivity was separated from free ligand by filtration through GF/C glass fiber filter plates (Millipore, Billerica, MA) pre-soaked for 1 h in polyethylenimine 0.2% at 4°C and washed three times with 170 µL of ice-cold washing buffer (50 mM Tris-HCl, pH 7.5, 0.2% bovine serum albumin). Receptor-bound radioactivity was measured in a γ-counter 1470 Wizard form PerkinElmer (80% counting efficiency). B_max_ was calculated using the formula B_max_ = IC_50_ (Bo/T) from the displacement studies, where IC_50_ represents the molar dose of Apelin-13 at which 50% of the bound tracer was displaced, Bo is the amount of cpm bound at equilibrium in the absence of unlabeled Apelin-13, and T is the total amount of cpm added in the incubation (Swillens, 1992). Dissociation K_i_ was calculated from the IC_50_ value using Cheng-Prusoff equation (Cheng and Prusoff, 1973), and all binding data were calculated and plotted using GraphPad Prism 8 (GraphPad Software Inc., CA, United States) and represented as the mean ± SEM of three independent experiments.

### Peptide Stability

Rat enzymes rACE2 and rNEP were purchased from Sino Biological (Beijing, China) while rACE was obtained from EMD Millipore (Burlington, MA, United States). Enzymes were dissolved in milli-Q water (at 1 µM for rACE2/rNEP and 1.65 µM for rACE), aliquoted and stored at −80°C, as recommended by suppliers. Buffer for enzymatic assay (100 mM Tris-HCl, pH 7.4, 100 mM NaCl, 10 µM ZnCl_2_) was prepared like previously described (McKinnie et al., 2016). First, 5 µL of Ape13 (1 mM) or Elabela (1 mM) was mixed with 5 µL of N,N-dimethylbenzamide (5 mM, internal standard) and diluted in 95 µL buffer. Before adding the enzyme, the blank sample (t = 0 min) was prepared by removing 10 µL of this mixture and quenched with 10 µL of EDTA 0.5 M. To initiate the enzymatic reaction, 5 µL of enzyme (1 µM or 1.65 µM) was added to the mixture (final enzyme concentration around 50 nM or 82 nM). This mixture was kept in an incubator at 37°C equipped with an orbital shaker (300 rpm). Ten µL was sampled at 5, 10, 20, 60, and 120 min from this mixture, and quenched with 10 µL EDTA 0.5 M (prepared in milli-Q water and filtered) to inactivate the enzyme. Samples were diluted with 40 µL water and analyzed using an UPLC-MS system from Waters (MA, United States) (Acquity UPLC^®^ Protein BEH C4 column (2.1 × 50 mm) packed with 1.7 µm particles, pore 300 Å) with the following gradient: acetonitrile and water with 0.1% HCOOH (0→0.2 min: 5% acetonitrile; 0.2→1.5 min: 5%→95%; 1.5→1.8 min: 95%; 1.8→2.0 min: 95%→5%; 2.0→2.5 min: 5%). First analyses (t = 0 min, t = 120 min) scanned m/z from 300–1200 to evaluate if peptides are cleaved and peptide fragments will be detected. After identifying the m/z of peptides and fragments, peptides were quantified using single ion mode (SIM). During the assay with rNEP, Elabela was partially oxidized (5–45% from t = 0 min to t = 120 min), both oxidized and non-oxidized products were measured to have a total amount of ELA. Angiotensin II was used as reference substrate in the assay with rACE2 and rNEP and angiotensin I was used as reference substrate in the assay with rACE. Experiments were repeated 3-5 times. Percentage of remaining peptide was plotted to an exponential decay curve using GraphPad Prism 8 (GraphPad Software Inc., CA, United States).

### Data and Statistical Analysis

Data are presented as mean values ± SEM. Comparisons of two experimental groups was determined using Student’s two-tailed *t*-test. Comparisons of parameters among more than two groups were analyzed by one-way analysis of variance (ANOVA) followed by Bonferroni’s correction for post hoc multiple comparisons when *F* ANOVA achieved *p* < 0.05, and there was no significant variance in homogeneity. Normal distribution and variance homogeneity were assessed by using the Shapiro-Wilk test. All statistics were performed using GraphPad Prism 8 (GraphPad Software Inc., CA, United States) and *p* < 0.05 was considered statistically significant.

## Results

### High-Salt Diet Alters the Apelinergic System in Hypertensive Rats

**FIGURE 1:**
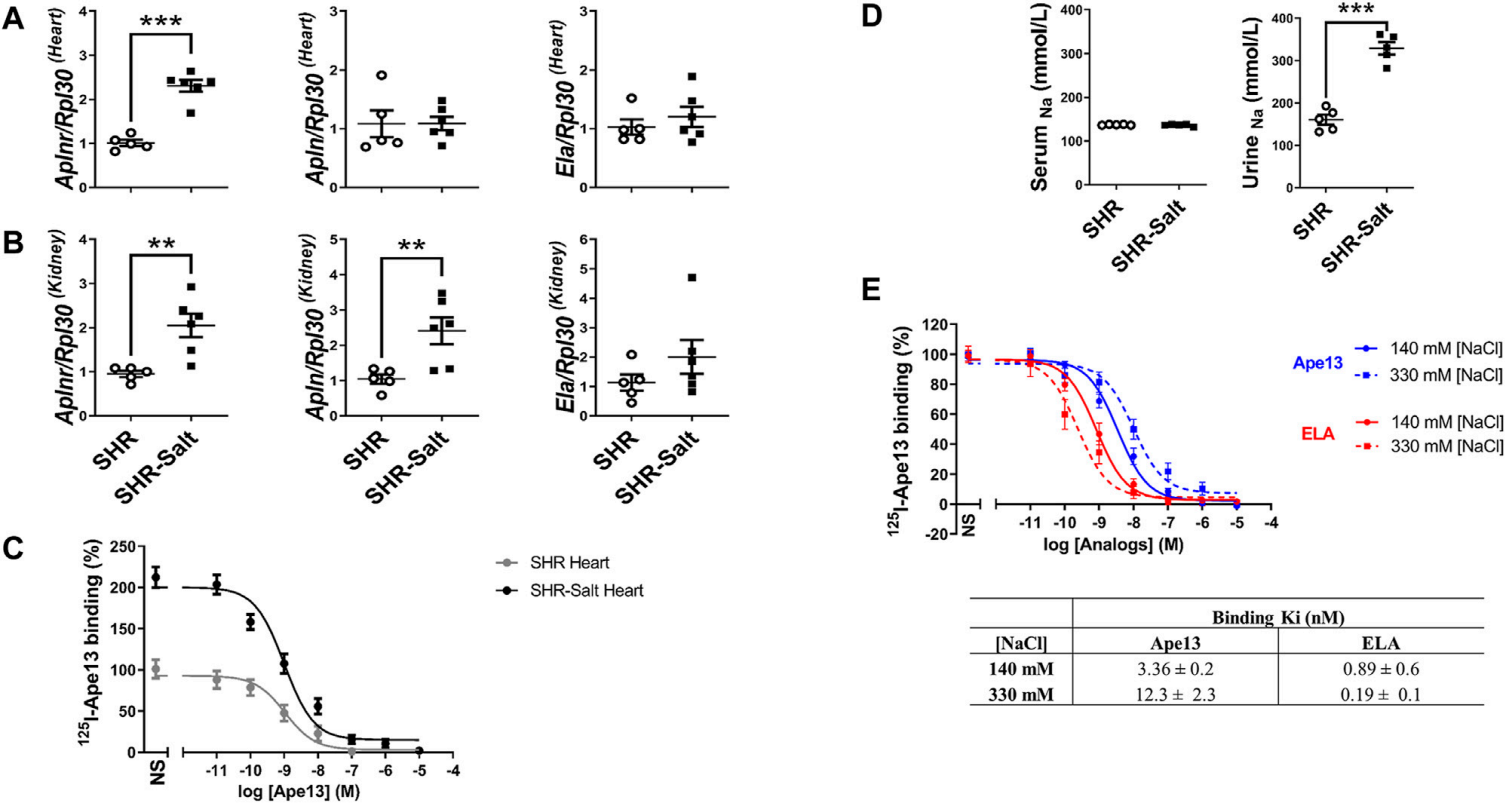
High-salt diet changes the expression of the apelinergic system in hypertensive rats. **(A,B)**, quantitative RT-PCR analysis of mRNA levels of APJ receptor (*Aplnr*) and its endogenous ligands Apelin-13 (*Apln*) and Elabela (*Ela*), normalized with the housekeeping gene Ribosomal protein L30 (*Rpl30*), in hearts **(A)** and kidneys **(B)** of SHR fed standard-(SHR) or high-salt (SHR-Salt) diets. All data are represented with individual values and means ± SEM. **p < 0.01, ***p < 0.001, the data were analyzed with Student’s two-tailed *t*-test; **(C)**, competition binding curves of Apelin-13 (Ape13) on total hearts of SHR and SHR-Salt. All values are means ± SEM of three independent experiments; **(D)**, measurement of sodium concentrations in serum and urine (SHR *n* = 5, SHR-Salt *n* = 6). All data are individual values with means ± SEM. ***p < 0.001, the data were analyzed with Student’s two-tailed *t*-test; **(E)**, competition binding curves of Ape13 and Elabela (ELA) in HEK293 stably expressing APJ at physiological (140 mM) or high (330 mM) NaCl concentrations. All values are means ± SEM of three independent experiments.

In order to evaluate the potential of APJ as a pharmacological target, we first carried out experiments with 10-week-old male spontaneously hypertensive rats (SHR) fed on a standard-(0.3% NaCl) or high-salt (8% NaCl) diet (Matavelli et al., 2007). After completing 6 weeks of the diet, the mRNA expression of APJ was significantly increased in heart and kidney of high-salt-treated SHR rats as compared to SHR rats fed with a standard-salt diet (Figures 1A,B). As shown by ligand-receptor binding assays, this upregulation was accompanied by increased APJ protein expression in the heart of SHR rats supplemented with a high-salt diet (Bmax: 54 ± 0.1 fmol/mg), compared with SHRs fed a standard-salt diet (Bmax: 7.3 ± 0.1 fmol/mg) (Figure 1C), without any change in Ki (0.55 ± 0.2 nM and 0.76 ± 0.1 nM, respectively). We also found that the mRNA expression of the endogenous ligand Ape13 was significantly upregulated in kidneys of SHR rats fed with high-salt diet, whereas only an upward trend in the expression of Elabela was observed (Figure 1B). Interestingly, beside *Apln* upregulation in kidneys, none of the apelinergic system’s genes were differentially expressed in the heart and kidneys of SHR compared with normotensive WKY adult rats (Supplementary Figure 1). Thus, these results suggest that high-salt intake *per se* increases the expression of APJ in heart and kidneys of SHR rats. To further assess the effect of high sodium intake on APJ receptor binding, we conducted competition binding studies with Ape13 and Elabela in the presence of varying sodium concentrations, previously determined based on sodium concentrations found in serum (140 mM [NaCl]) and urine (330 mM [NaCl]) of SHR-rats receiving high-salt diet (Figure 1D). We observed a sodium-dependent dichotomic effect on Ape13 and Elabela binding affinities for APJ (Figure 1E). Indeed, when sodium concentration is increased to 330 mM, an improvement in the Elabela affinity can be measured (Ki_330nM_: 0.19 ± 0.1 nM vs. Ki_140nM_: 0.89 ± 0.6 nM), whereas it is the reverse for Ape13 (Ki_330nM_: 12.3 ± 2.3 nM vs. Ki_140nM_: 3.36 ± 0.2 nM). Overall, these results indicate that the apelinergic system is salt-sensitive, with upregulation of expression in key pressure-regulating organs. In addition, Elabela appears to be more effective than Ape13 in binding APJ under high-salt conditions.

### Continuous ELA Infusion Alleviates Cardiovascular Dysfunction and Remodeling Induced by Dietary Salt Intake and Hypertension

**FIGURE 2:**
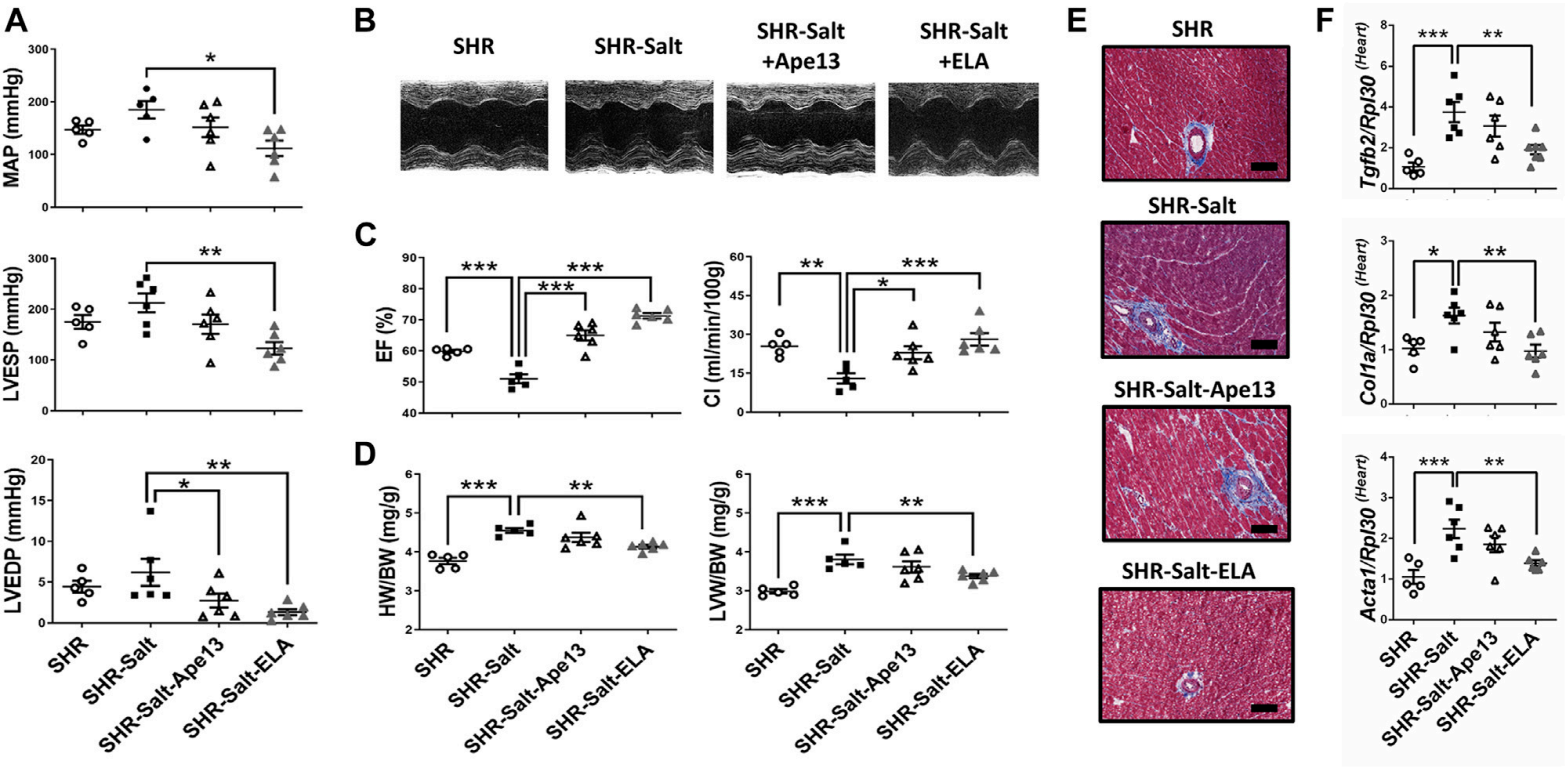
Chronic Elabela infusion alleviates cardiovascular dysfunction and remodeling induced by high-salt diet in hypertensive rats. **(A)**, cardiac function measurements in SHR rats fed with standard-(SHR) or high-salt diet (8% of NaCl) and treated with either Apelin-13 (SHR-Salt-Ape13), Elabela (SHR-Salt-ELA) or saline (SHR-Salt). Mean arterial pressure (MAP), left ventricular end-systolic (LVESP) and end-diastolic pressures (LVEDP) were measured with a pressure catheter; **(B)**, representative images of short axis M-mode echocardiography after 6 weeks of diet regimen; **(C)**, measurement of the ejection fraction (EF%) and cardiac index (CI); **(D)**, heart weight to body weight ratio (HW/BW) and left ventricular weight to body weight ratio (LVW/BW); **(E)**, representative histology of heart stained with Masson’s trichrome (Scale bars: 100 μm); **(F)**, quantitative RT-PCR analysis of pro-fibrosis mRNA levels of transforming growth factor-β2 (*Tgfb2*), collagen 1a (*Col1a*), and skeletal muscle α1-actin (*Acta1*) in hearts. The Ribosomal protein L30 (*Rpl30*) was used as a reporter gene. Data were normalized to SHR rats fed with standard-salt diet (0.3% of NaCl). (SHR *n* = 5, SHR-Salt *n* = 6, SHR-Salt-Ape13 *n* = 6, and SHR-Salt-ELA *n* = 6). All data are individual values with means ± SEM. *p < 0.05, **p < 0.01, ***p < 0.001, the data were analyzed with one-way ANOVA and Bonferroni’s post hoc test.

We next investigated the cardiovascular effects of a continuous 6-week infusion of equimolar doses (10 nmol/kg/hr) of Ape13, the predominant Apelin fragment detected in circulation (Zhen et al., 2013), or Elabela in SHR rats. Interestingly, we observed abolition of hypertension induced by a high-salt diet after Elabela treatment, while Ape13 had no significant effect (Figure 2A). This cardioprotective effect of Elabela on hypertension results in a significant decrease in mean arterial pressure (MAP) and left ventricular end-systolic (LVESP), suggesting reduced afterload in salt-loaded SHR animals (Figures 2A,B). In addition, Elabela treatment and to a lower extent Ape13 significantly improved cardiac function impaired by the high-salt diet, resulting in recovery of fractional shortening (FS), cardiac index (CI), and ejection fraction (EF) (Figures 2B,C; Table 1). Similarly, sustained Elabela infusion protected salt-loaded SHR animals from cardiac hypertrophy, as evidenced by the significant decrease of LV anterior and posterior wall thickness as well as heart weight (HW/BW) and left ventricular weight (LVW/BW) (Figure 2D; Table 1). Interestingly, assessment of these cardiovascular parameters in normotensive WKY rats compared to SHRs suggests that chronic treatment for 6 weeks with Ape13 or Elabela does not prevent or reverse the cardiovascular damage caused by hypertension *per se* in SHR rats receiving daily normal-salt intake (Supplementary Figure 2). Furthermore, reduced LV end-diastolic pressures (LVEDP) and aortic pulse propagation velocity indicate that Elabela alleviated the LV and aortic stiffness in salt-loaded SHR animals. Moreover, histological analysis revealed that the high sodium diet was responsible for the appearance of areas of cardiac fibrosis in the perivascular regions of SHR hearts (Figure 2E). Consistently, Elabela treatment markedly downregulated the expression of pro-fibrotic collagen 1a (*Col1a*), skeletal muscle alpha-1 actin (*Acta1*), and transforming growth factor-β2 (*Tgfb2*) genes, which were significantly increased in the heart of salt-treated SHR rats (Figure 2F). No changes in animal body weight or heart rate were observed after agonist treatment (Table 1). Taken together, these results demonstrate that chronic exogenous Elabela treatment can protect salt-loaded SHR hearts from fibrosis and reveal the protective effects of Elabela on pressure overload, cardiovascular dysfunction and remodeling.

### Sustained Infusion of Elabela Protects Salt-Loaded Spontaneously Hypertensive Rats From Renal Dysfunction and Remodeling

**FIGURE 3:**
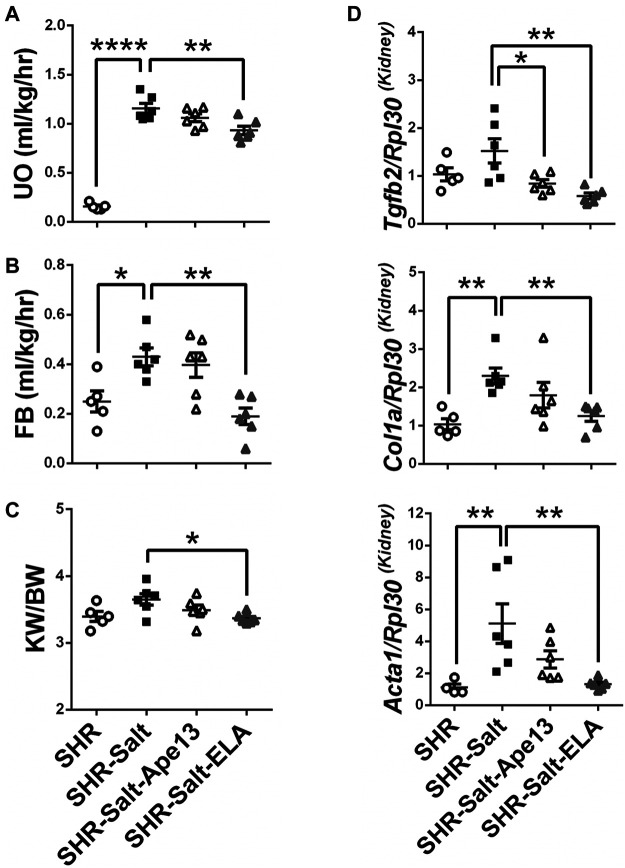
Chronic Elabela infusion protects salt-load hypertensive rats from renal dysfunction and remodeling. **(A,B)**, fluid homeostasis of SHR rats fed with standard- (SHR) or high-salt diet (8% of NaCl) and treated with either Apelin-13 (SHR-Salt-Ape13), Elabela (SHR-Salt-ELA) or saline (SHR-Salt). Fluid balance (FB) was calculated by the volume of the urinary output (UO) subtracted from the water intake measured after 24 h in the metabolic cage; **(C)**, kidney weight to body weight ratio (KW/BW); **(D)**, quantitative RT-PCR analysis for expression of pro-fibrosis genes. mRNA levels of transforming growth factor-β2 (*Tgfb2*), collagen 1a (*Col1a*), and skeletal muscle alpha-1 actin (*Acta1*) were measured in kidneys. The Ribosomal protein L30 (*Rpl30*) was used as a reporter gene. Data were normalized with SHR rats fed with standard-salt diet (0.3% of NaCl). (SHR *n* = 5, SHR-Salt *n* = 6, SHR-Salt-Ape13 *n* = 6, and SHR-Salt-ELA *n* = 6). All data are individual values with means ± SEM. *p < 0.05, **p < 0.01, ****p < 0.0001, the data were analyzed with one-way ANOVA and Bonferroni’s post hoc test.

There is growing evidence demonstrating that severe myocardial fibrosis and biventricular dysfunction are associated with renal hemodynamic dysfunction in salt-loaded SHRs (Varagic et al., 2006; Matavelli et al., 2007). Accordingly, salt excess produced detrimental effects on renal function in SHR rats (Figures 3A–C). Importantly, only exogenous Elabela significantly restored renal function and fluid homeostasis in severe hypertensive rats, resulting in improved urinary output (UO), fluid balance (FB) and protection from kidney hypertrophy (Figures 3A–C). As observed in diseased heart, expression of the pro-fibrotic *Col1a*, *Acta1*, and *Tgfb2* genes in kidneys were significantly increased in SHR rats placed on an 8% salt diet. Consistently, chronic infusion of Elabela markedly decreased the expression of these pro-fibrotic genes, while Ape13 only reduced the *Tgfb2* mRNA level (Figure 3D). Altogether, these results indicate that Elabela is more effective than Ape13 in protecting salt-loaded hypertensive rats from renal dysfunction, hypertrophy, and remodeling.

### Chronic Elabela Infusion Regulates the Renin-Angiotensin-Aldosterone System in the Heart and Kidneys of Salt-Loaded Hypertensive Rats

**FIGURE 4:**
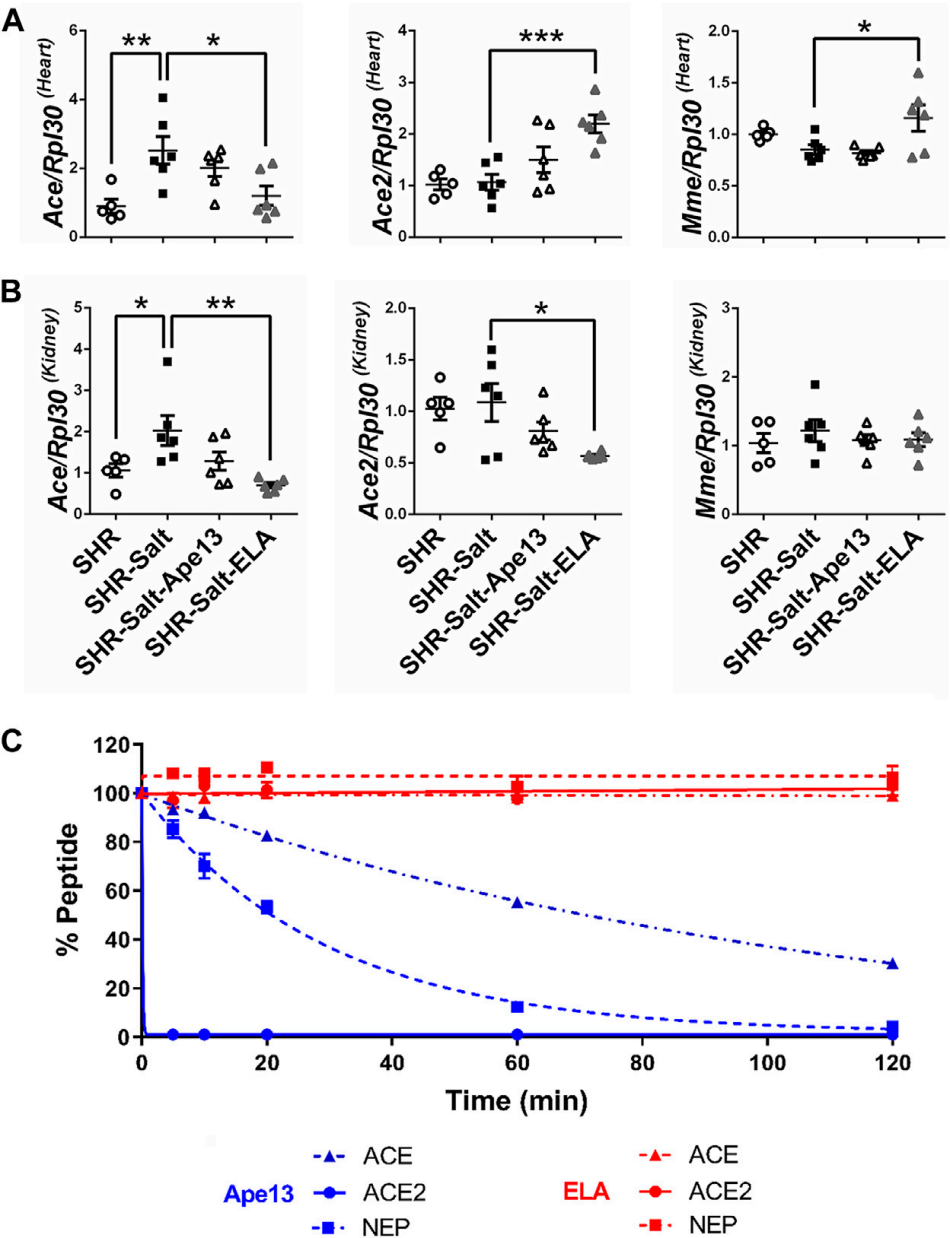
Chronic Elabela infusion regulates the renin-angiotensin-aldosterone system in heart and kidneys of salt-loaded hypertensive SHR. **(A,B)**, quantitative RT-PCR analysis of RAAS genes. mRNA levels of angiotensin converting enzyme (*ACE*), angiotensin converting enzyme 2 (*ACE2*), ratio *ACE/ACE2*, and neprilysin (*Mme*) in heart **(A)** and kidneys **(B)** of SHR rats fed with standard-(SHR) or high-salt diet (8% of NaCl) and treated with either Apelin-13 (SHR-Salt-Ape13), Elabela (SHR-Salt-ELA) or saline (SHR-Salt). The Ribosomal protein L30 (*Rpl30*) of heart and kidneys was used as a reporter gene and the data normalized to SHR rats fed with standard-salt diet (0.3% of NaCl). All data are individual values with means ± SEM. *p < 0.05, **p < 0.01, the data were analyzed with one-way ANOVA and Bonferroni’s post hoc test (SHR *n* = 5, SHR-Salt *n* = 6, SHR-Salt-Ape13 *n* = 6, and SHR-Salt-ELA *n* = 6); **(C)**, cleavage assay of 1 mM of Ape13 or ELA with 50 nM of purified rat angiotensin converting enzyme (ACE), angiotensin converting enzyme 2 (ACE2) or neprilysin (NEP). All values are means ± SEM of three independent experiments.

The renin-angiotensin-aldosterone system (RAAS) is a key pathway involved in the regulation of fluid balance and blood pressure, both of which are altered in hypertensive salt-loaded rats. Moreover, it has been suggested that RAAS overactivation plays a crucial role in heart failure progression in rodents and promotes the negative effects of salt excess on heart and kidneys (Susic et al., 2009). Here, we observed that the high-salt diet in SHR rats led to a significant increase in angiotensin converting enzyme (*Ace*) mRNA expression, but not angiotensin converting enzyme 2 (*Ace2*) and neprilysin (*Mme*), both in the heart and kidneys (Figures 4A,B). Unlike Ape13 treatment, chronic infusion of Elabela had differential effects on the expression of these enzymes. In heart, Elabela counteracted the effect of high-salt exposure, normalizing the gene expression of ACE, while ACE2 and neprilysin mRNA levels were significantly increased compared to salt-loaded hypertensive rats (Figure 4A). In the kidney, the increase in ACE expression in salt-loaded SHR rats was completely abolished after Elabela treatment, while neprilysin was not affected (Figure 4B). Finally, RAAS enzymes are known to degrade, regulate or inactivate apelin peptides (Vickers et al., 2002; McKinnie et al., 2016). To investigate the activity of RAAS proteases on the Elabela peptide, we compared the proteolytic activity of those purified enzymes on apelinergic ligands. Interestingly, ACE, ACE2, and neprilysin were all unable to cleave Elabela within 2 h, while Ape13 was rapidly and fully hydrolyzed by ACE2 and neprilysin and only partially by ACE (Figure 4C). Altogether, these data suggest that in addition to being resistant to RAAS enzyme degradation, Elabela may reduce the RAAS overactivation in the heart and kidneys in the pathogenesis of salt-induced hypertension.

## Discussion

The main purpose of this study was to investigate if the Ape13-Elabela-APJ receptor axis may represent a therapeutic target for improving severe hypertension exacerbated by dietary high-salt intake. Similar to humans, SHR rats exhibit reduced cardiac output and increased total peripheral vascular resistance (Okamoto and Aoki, 1963; Ferrone et al., 1979; Doggrell and Brown, 1998). As observed in humans, the severity of hypertension in rats is worsened by excessive sodium intake, thereby exacerbating the pathological condition, such as increased blood pressure and cardiorenal dysfunction (Ahn et al., 2004; Matavelli et al., 2007; Whelton et al., 2012). Here, we demonstrate for the first time that Elabela is more effective than Ape13 in reducing high blood pressure, cardiorenal dysfunctions, fibrosis, and hypertrophy induced by a 6-week dietary high-salt intake in male SHR rats. Interestingly, these beneficial effects observed after continuous infusion of Elabela were associated with a counter-regulatory role of the ACE/ACE2/neprilysin axis of the renin-angiotensin-aldosterone system in heart and kidneys of salt-loaded male SHR rats. The question of whether this is true in hypertensive female rats fed a high-salt diet still needs to be considered in view of the sexual dimorphism of hypertension, sex differences in the expression of RAAS components, and the significant impact of female sex hormones on salt-sensitive hypertension (Yanes et al., 2006; Sullivan et al., 2010; Bubb et al., 2012). Nonetheless, we highlighted the potent protective action of Elabela against salt-induced heart and kidney damage.

We first characterized the severity of hypertension and cardiorenal dysfunction in SHR rats fed high-salt diet (8% of NaCl) relative to SHRs on normal-salt chow (0.3% of NaCl). Consistent with previous reports (Varagic et al., 2006; Susic et al., 2010), the high-salt challenge was responsible for a significant collapse of cardiovascular and renal functions well supported by increased hypertrophy and fibrosis in heart and kidneys. This excessive sodium intake leading to severe hypertension was accompanied by a significant upregulation in the expression of APJ in both heart and kidneys. Apart from Ape13 in kidneys, mRNA expression of both endogenous ligands was not altered in these high-salt-treated SHR rats. This increase in the expression levels of APJ in salt-loaded SHR was associated with significant beneficial effects of Elabela and to a lesser extent of Ape13 on cardiac function, notably with an improvement in cardiac index and ejection fraction. Importantly, only Elabela reduced the high blood pressure, cardiac hypertrophy and fibrosis induced by excessive salt consumption. It would be very interesting to determine if the expression of APJ is also dependent on daily salt intake in salt-sensitive Dahl rats *vs* salt-resistant rats. Given some of the differences between SHR and salt-sensitive Dahl rats, such as renal metabolism and intermittent baroreflex dysfunction (Gu et al., 2020; Tian and Liang, 2021), future comparative studies of the effectiveness of Elabela in these murine models could provide mechanistic insights about the potential signaling pathways involved in the beneficial effects of Elabela on salt-induced cardiovascular and renal injury.

Since its discovery as the second endogenous ligand of the APJ receptor (Szokodi et al., 2002), Elabela has received growing interest given the pharmacological role of the Apelin-APJ system in a variety of physiological and pathological processes, which include cardiovascular and kidney diseases, but also metabolic disorders, such as insulin resistance/type 2 diabetes and obesity, cancer development and progression, ischemic stroke as well as neurodegenerative diseases (Scimia et al., 2012; Chen et al., 2018; Coquerel et al., 2018; Wysocka et al., 2018; Castan-Laurell et al., 2019; Huang et al., 2019; Kinjo et al., 2020; Luo et al., 2020; Masoumi et al., 2020; Tian et al., 2020). In particular, similar to Ape13, Elabela which binds to APJ and activates the β-arrestin-2 and G_αi_ signaling pathways exerts protective effects against various cardiovascular diseases and related complications (Coquerel et al., 2018; Kuba et al., 2019; Liu et al., 2019; Chen et al., 2020a; Liu et al., 2020; Ma et al., 2020; Xu, 2020). For instance, systemic administration of Elabela has been shown to improve cardiac function following acute myocardial infarction and derived cardiovascular events, including heart failure, myocardial ischemia/reperfusion (I/R) injury, arrhythmias, and stroke (Sato et al., 2017; Pan et al., 2020; Yu et al., 2020). Consistently, a link between hypertension and Elabela defect has been observed in pulmonary arterial hypertension (PAH) in both rats and humans (Yang et al., 2017). Also known for its role in the development of fetal heart and blood vessels (Chng et al., 2013; Chen et al., 2020a), release of Elabela by the placenta also prevents the pathogenesis of preeclampsia, a severe form of gestational hypertension, by promoting placental angiogenesis (Ho et al., 2017; Liu et al., 2019). Finally, activation of the Elabela-APJ receptor axis has recently been proposed as an alternative to the recommended catecholamines for supporting polymicrobial sepsis-induced inflammatory myocardial dysfunction (Chagnon et al., 2017; Coquerel et al., 2017).

In addition to its cardioprotective effect, the Ape13-Elabela-APJ receptor system plays an important role in renal physiology, notably in the regulation of fluid balance (Flahault et al., 2017). Indeed, the APJ receptor is expressed in the kidney and Ape13 has been found to exert vasoactive effects on afferent and efferent arterioles and to regulate the activity of the aquaporin water channel AQP2, thus controlling the diuresis, urine osmolality, and renal hemodynamic function (Hus-Citharel et al., 2008; Roberts et al., 2009; Hus-Citharel et al., 2014; Chen et al., 2015; Boulkeroua et al., 2019). Unlike the wide tissue distribution of APJ and Ape13, Elabela is mainly expressed in the kidneys, especially in the inner medulla collecting duct and also acts as an aquaretic agent (Deng et al., 2015; Wang et al., 2015b; O’Carroll et al., 2017). Interestingly, Elabela has recently been shown to be better than Ape13 in improving fluid homeostasis and in limiting renal dysfunction in various kidney diseases, such as diabetic nephropathy, autosomal dominant polycystic kidney disease, renal ischemia/reperfusion injury, renal fibrosis, and septic shock (Day et al., 2013; Lacquaniti et al., 2013; Zhang et al., 2013; Chen et al., 2017; Coquerel et al., 2017; O’Carroll et al., 2017; Huang et al., 2018; Zhang et al., 2018). Here, we found that chronic infusion of Elabela is more effective than Ape13 in protecting salt-loaded hypertensive rats from renal dysfunction, kidney hypertrophy, and remodeling. Accordingly, Elabela treatment was recently reported to preserve the glomerular structure, to prevent renal fibrosis and to block the expression of fibrosis-related genes in the kidneys of Dahl salt-sensitive rats on a high-salt diet and deoxycorticosterone acetate/salt-treated rats (Schreiber et al., 2017; Chen et al., 2020b; Xu et al., 2020). Consistently, recent clinical data have shown, in contrast to Ape13, that the decrease in serum Elabela levels is also strongly associated with deterioration of renal function and worsening stages of chronic kidney disease (Lu et al., 2020).

It is well known that the progression of heart failure is closely linked to the renin-angiotensin-aldosterone system and that the pharmacological modulation of RAAS by ACE inhibitors, aldosterone and angiotensin II (Ang II) type 1 receptor antagonists, or direct renin inhibitors can be beneficial for managing patients with hypertension and heart failure (McMurray et al., 2014; Solomon et al., 2017; Cosentino et al., 2019). In stressed heart, ACE is up-regulated inversely to ACE2, leading to an imbalance in the ACE/ACE2 ratio, increased production of Ang II, and disturbance in RAAS homeostasis, which promote Ang II-induced hypertension, cardiac hypertrophy, and fibrosis (Wang et al., 2015a; Yang et al., 2016). Consequently, vasodilatation and cardiorenal protective effects can be achieved with a shift from the detrimental ACE-Ang II-AT1R axis towards the activation of the ACE2-Ang (1-7)-Mas receptor axis (Iwai and Horiuchi, 2009; Mizuiri and Ohashi, 2015; Domenig et al., 2016). After 6 weeks of high-salt diet, we observed a significant upregulation of ACE expression in SHR heart. However, chronic treatment with Elabela counteracted this deleterious effect by downregulating ACE mRNA levels and by increasing cardiac ACE2 and neprilysin gene transcriptions, thereby possibly promoting the cardioprotective action of the ACE2-Ang (1-7)-Mas axis in SHR salt-loaded rats. Our results support previous findings demonstrating the opposing action and physiological crosstalk between the Ape13-APJ and Ang II-AT1R systems in regulating the cardiovascular function (Chatterjee et al., 2020). Indeed, while an increased vasopressor response to Ang II is found in APJ knockout mice, ACE2 is downregulated in apelin-deficient mice (Ishida et al., 2004; Sato et al., 2013). Likewise, Ape13 binding to APJ induces an upregulation of ACE2 expression in pathological hearts, increases the conversion of Ang II into Ang (1–7) and antagonizes the Ang II-AT1R signaling by promoting APJ-AT1R heterodimerization (Sato et al., 2013; Siddiquee et al., 2013). Finally, Elabela was demonstrated to antagonize Ang II-induced hypertension and cardiac damage, as shown here in high-salt-treated SHR rats (Sato et al., 2017).

In kidneys, increases in ACE/ACE2 ratio induced via the Ang II-AT1R axis have a significant influence on the development of severe renal damage and disease progression, such as diabetes, nephropathy and hypertension in humans (Wakahara et al., 2007; Mizuiri et al., 2008). In addition, it should be pointed that a diet high in salt enhances ACE activity in healthy normotensive rats and then alters the ACE/ACE2 balance leading to kidney damage (Hamming et al., 2008; Crestani et al., 2014; Mizuiri and Ohashi, 2015). Then, after 6 weeks of high daily salt intake, while it was maintained at a physiological concentration in serum, we observed a pronounced increase in sodium concentration in urine correlated with an increase expression of ACE in the kidneys. It has been demonstrated that intrarenal activation of RAAS can regulate blood pressure and water retention and operate independently of the systemic RAAS (Samuel et al., 2012), which could in part explain the differences in RAAS regulation observed between heart and kidneys. Importantly, our results reveal that chronic infusion of Elabela but not of Ape13 restores ACE expression in kidneys of salt-loaded SHR rats, without affecting the expression of neprilysin. These results are in agreement with recent findings showing that administration of exogenous Elabela to high-salt-loaded Dahl salt-sensitive rats is able to antagonize the intrarenal RAAS in the distal nephron, thereby resulting in lower blood pressure and protection against renal injury (Xu et al., 2020).

Apelin-13 has been reported to be one of the most potent endogenous inotrope positive agents of myocardial contractility (Szokodi et al., 2002; Perjes et al., 2016). We previously demonstrated that Elabela is superior to Ape13 in improving hemodynamics in isolated healthy rat hearts, suggesting that the two endogenous APJ ligands may exert cardio-protective effects with different potencies (Coquerel et al., 2017; Coquerel et al., 2018). To better understand how Elabela is more effective than Ape13 in treating the blood pressure raising effects caused by excessive daily salt intake, we also investigated the impact of sodium on ligand-receptor interaction. It has been reported that several class AG protein-coupled receptors, including APJ, share a sodium binding pocket that can allosterically affect the orthosteric ligand binding (Katritch et al., 2014; Ma et al., 2017). To mimic the sodium conditions in heart and kidneys of salt-loaded SHR rats, we then compared the *in vitro* affinity of Elabela and Ape13 for APJ at sodium concentrations measured in serum (i.e., 140 mM) and urine (i.e., 330 mM) from those hypertensive rats. Interestingly, the increased sodium concentrations appear to promote the binding of Elabela to APJ. At 330 mM sodium, corresponding to a state of sodium stress in kidney, APJ preferentially binds Elabela over Ape13 and then could partly explain why chronic infusion of Elabela is more effective to reduce intrarenal pressure and limits cardiorenal dysfunctions and fibrotic remodeling in salt-loaded SHR rats. Our results also indicated that the high-salt diet disrupts the balance between the opposing components of RAAS in the heart and kidneys of severely hypertensive rats. Importantly, the beneficial effects of apelin peptides are tightly regulated in a negative feedback loop by the increased activity of ACE2. Indeed, the action of apelin peptides is limited due to their rapid degradation by proteases, including ACE2 and neprilysin (Vickers et al., 2002; McKinnie et al., 2016). However, there is no information regarding the enzymatic activities of RAAS towards ELA. Then, we have compared the kinetics of hydrolysis of the endogenous ligands Elabela and Ape13 by the ACE, ACE2, and neprilysin purified enzymes. Surprisingly, none of these RAAS enzymes degrade ELA, while Ape13 is fully hydrolyzed by ACE2 and neprilysin and, shown for the first time, partially by ACE. This increased stability of Elabela with respect to ACE, ACE2 and neprilysin constitutes further evidence of its improved efficacy compared to Ape13 in these salt-treated hypertensive rats. These results also open new questions about the pharmacokinetics and mechanism of action of Elabela in salt-sensitive hypertension, which may be unique to this APJ ligand, and require further investigation.

In summary, our results demonstrate that the Elabela-APJ receptor axis is a key counter-regulator of the RAAS in salt-loaded SHR rats. In addition to being resistant to RAAS enzymes, Elabela could indeed restore the ACE/ACE2 balance and exert cardiorenal protective effects against salt-driven hypertension and kidney damage. In particular, activation of the Elabela-APJ axis has been found to regulate the myocardial contractility, to control the fluid homeostasis and to exert antihypertensive, vasodilatory, anti-fibrotic and anti-remodeling effects. Altogether, these findings highlight that the sustained released of Elabela may serve as a novel therapy for the management of hypertension among patients at high risk for cardiovascular and chronic kidney diseases.

## Data Availability

The original contributions presented in the study are included in the article/Supplementary Material, further inquiries can be directed to the corresponding authors.

## Abbreviations

APJ, apelinergic receptor; SHR, spontaneously hypertensive rats; ACE, angiotensin conversion enzyme; RAAS, renin-angiotensin-aldosterone system; Ape13, apelin-13; ELA, Elabela; AT1R, angiotensin II type 1 receptor.
