# Supplementary material for: Elabela Protects Spontaneously Hypertensive Rats From Hypertension and Cardiorenal Dysfunctions Exacerbated by Dietary High-Salt Intake
Source: Front Pharmacol. 2021 Jul 27;12:709467. doi: 10.3389/fphar.2021.709467 (PMC8353398; doi:10.3389/fphar.2021.709467)
Supplement: Supplementary file 1 [file DataSheet1.pdf]

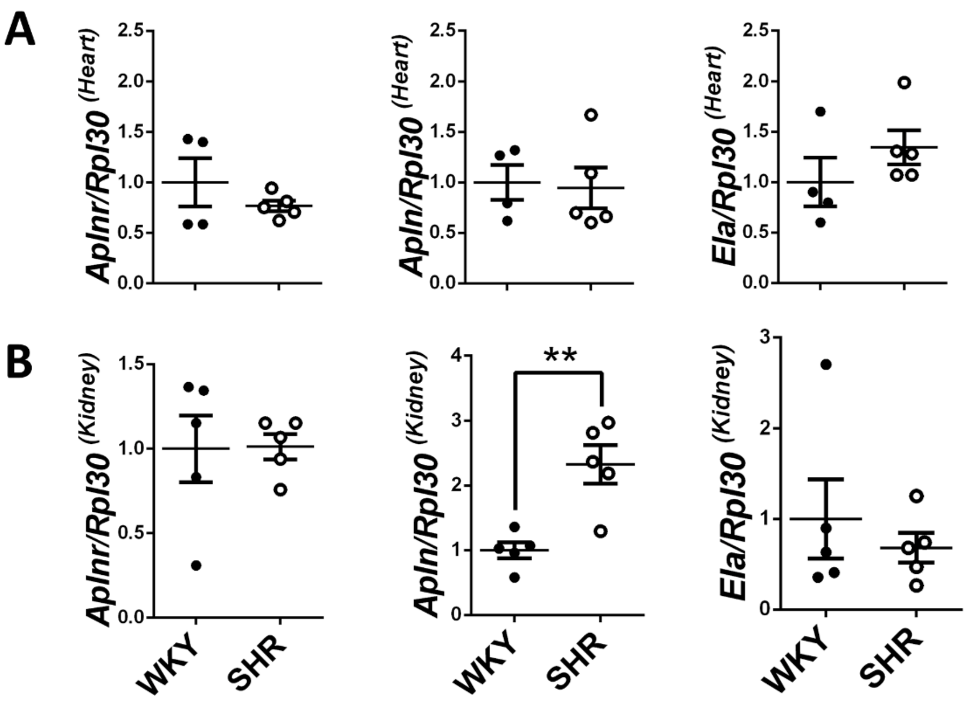

SUPPLEMENTAL FIGURE 1. Hypertension increases the Apelin mRNA expression in the kidney of adult male rats. (A and B), quantitative RT-PCR analysis of mRNA levels of APJ receptor (*Aplnr*) and its endogenous ligands Apelin-13 (*Apln*) and Elabela-32 (Ela*),* normalized with the housekeeping gene Ribosomal protein L30 (*Rpl30*), in hearts **(A)** and kidneys **(B)** of normotensive Wistar Kyoto (WKY) and spontaneously hypertensive rats (SHR) fed standard-salt diet (0.3% of NaCl). All data are represented with individual values and means ± SEM. **P<0.01, the data were analyzed with Student's two-tailed t-test.

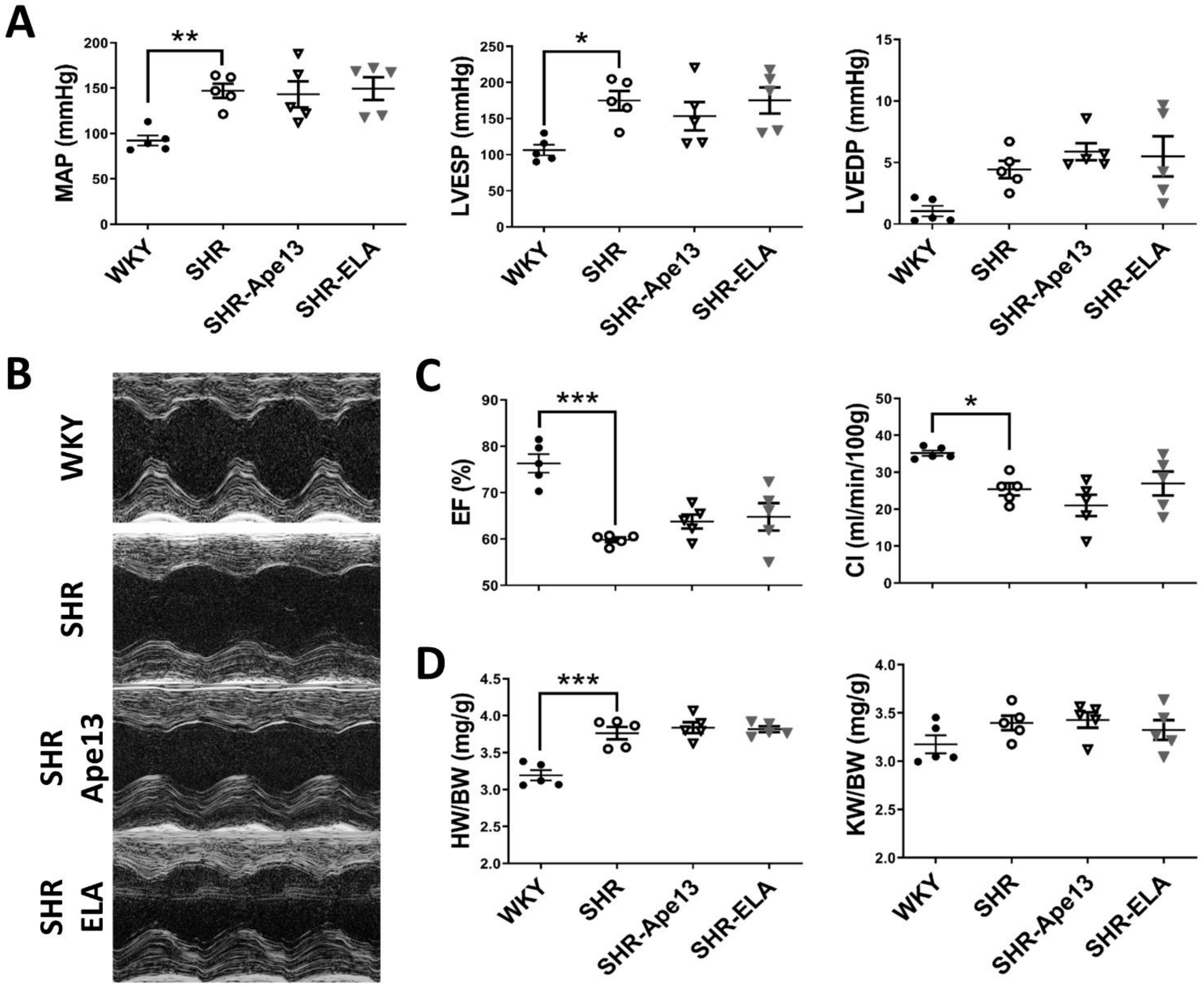

SUPPLEMENTAL FIGURE 2. Chronic activation of the apelinergic system does not alleviate the cardiovascular dysfunction and remodeling in adult male hypertensive rats. (A), cardiac function measurements in normotensive Wistar Kyoto (WKY) and SHR rats fed with standard-salt diet (0.3% of NaCl) and treated with either Apelin-13 (SHR-Ape13), Elabela-32 (SHR-ELA) or saline (SHR). Mean arterial pressure (MAP), left ventricular end-systolic (LVESP) and end-diastolic pressures (LVEDP) were measured with a pressure catheter; **(B)**, representative images of short axis M-mode echocardiography after 6 weeks of diet regimen; **(C)**, measurement of the ejection fraction (EF%) and cardiac index (CI); **(D)**, heart weight to body weight ratio (HW/BW) and kidney weight to body weight ratio (KW/BW). All data are individual values with means ± SEM. *P<0.05, **P<0.01, ***P<0.001, the data were analyzed with one-way ANOVA and Bonferroni’s post hoc test.

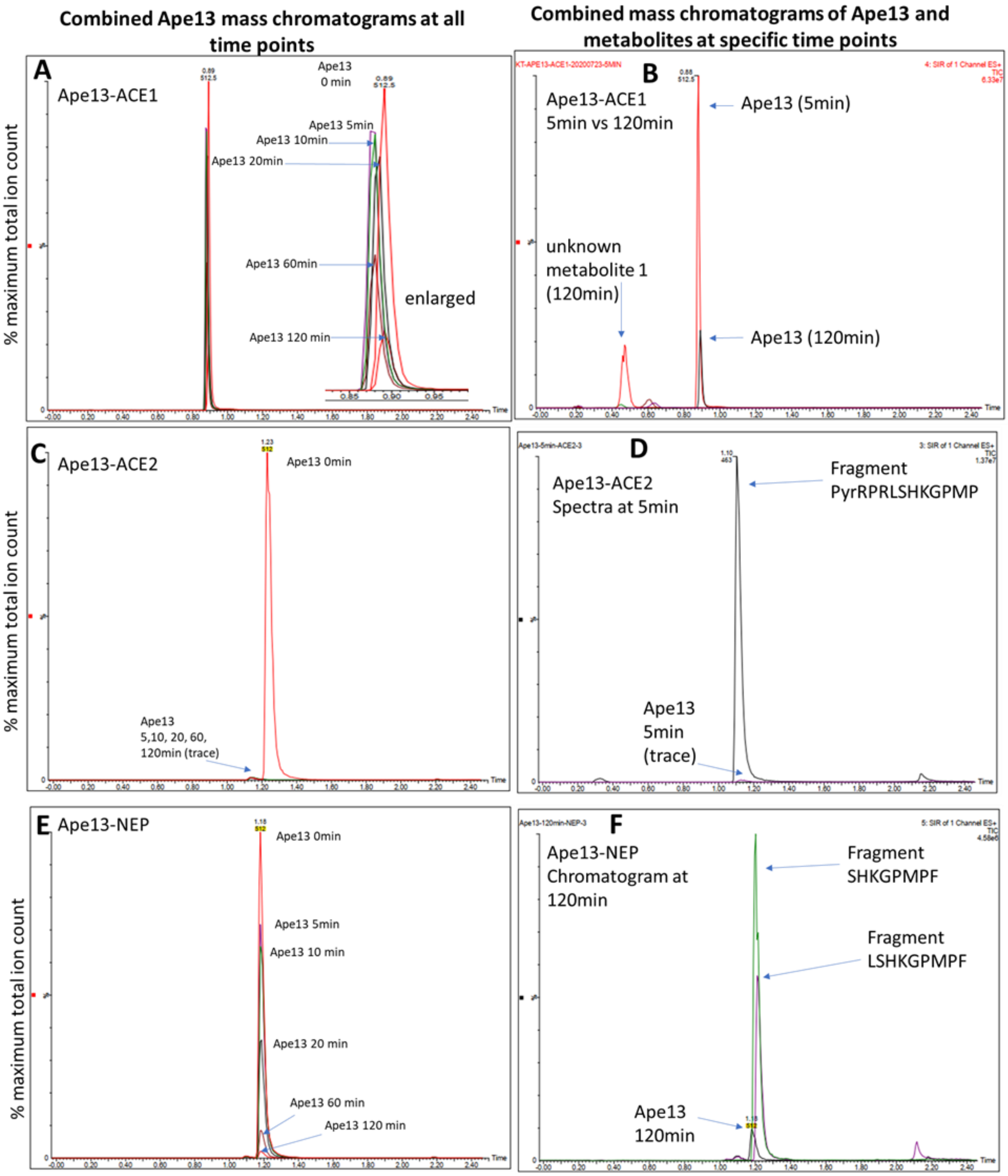

SUPPLEMENTAL FIGURE 3. Representative chromatograms of Apelin-13 *in vitro* metabolism. **(A)**, ACE1 cleaves Apelin-13 (Ape13). The superposition of the mass chromatograms of Ape13 m/z 512 at 0, 5, 10, 20, 60, 120 min shows a progressive degradation of Ape13 in the presence of ACE1; **(B)**, the chromatogram of Ape13 and its metabolites in the presence of ACE1 at 5 and 120 min; **(C)**, ACE2 rapidly cleaves Ape13. The overlay of the mass chromatograms of Ape13 m/z 512 at 0, 5, 10, 20, 60, 120 min shows complete degradation of Ape13 in the presence of ACE2 after 5 min; **(D)**, the chromatogram of Ape13 and its metabolites (m/z 463) in the presence of ACE2 at 5 min; **(E)**, NEP cleaves Ape13. The superposition of the mass chromatograms of Ape13 m/z 512 at 0, 5, 10, 20, 60, 120 min shows a gradual degradation of Ape13; **(F)**, the chromatogram of Ape13 and its metabolites (m/z 450 and m/z 507) in the presence of NEP at 120 min.

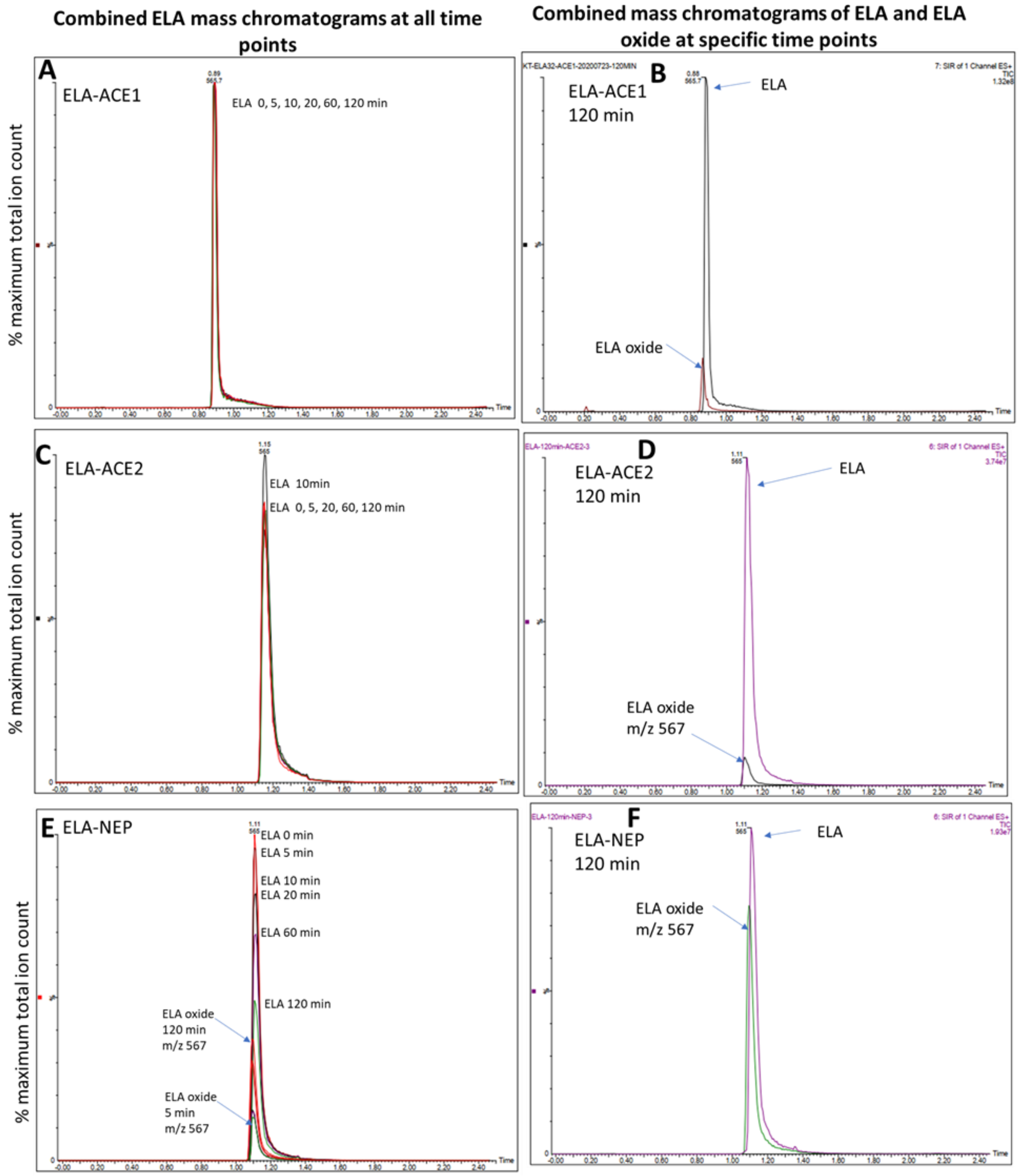

SUPPLEMENTAL FIGURE 4. Representative chromatograms of Elabela *in vitro* metabolism. (A), ACE1 does not cleave Elabela-32 (ELA). Overlay of the mass chromatograms of ELA m/z 565 at 0, 5, 10, 20, 60, 120 min shows no major change in ELA concentration in the presence of ACE1; **(B)**, the chromatogram of ELA and ELA oxide at 120 min in the presence of ACE1 shows just a small amount of ELA oxide; **(C)**, ACE2 does not cleave ELA. The superposition of the mass chromatograms of ELA m/z 565 at 0, 5, 10, 20, 60, 120 min shows no major change in ELA concentration in the presence of ACE2; **(D)**, the chromatogram of ELA and ELA oxide at 120 min in the presence of ACE2 shows just a small amount of ELA oxide; **(E)**, NEP does not cleave ELA. Overlay of the mass chromatograms of ELA m/z 565 at 0, 5, 10, 20, 60, 120 min shows a reduction in ELA concentration. However, this reduction is accompanied by an increasing amount of ELA oxide. When combined ELA and ELA oxide, the result shows that the total amount of ELA is unchanged; **(F)**, The chromatogram of ELA and ELA oxide at 120 min in the presence of NEP shows a significant amount of ELA oxide.
